# Cryo-EM structure of a transthyretin-derived amyloid fibril from a patient with hereditary ATTR amyloidosis

**DOI:** 10.1038/s41467-019-13038-z

**Published:** 2019-11-01

**Authors:** Matthias Schmidt, Sebastian Wiese, Volkan Adak, Jonas Engler, Shubhangi Agarwal, Günter Fritz, Per Westermark, Martin Zacharias, Marcus Fändrich

**Affiliations:** 1 Institute of Protein Biochemistry, Ulm University, 89081 Ulm, Germany; 2 Core Unit Mass Spectrometry and Proteomics, Ulm University, 89081 Ulm, Germany; 3 Institute of Microbiology, University of Hohenheim, 70599 Stuttgart, Germany; 4 Institute for Neuropathology, Faculty of Medicine, University of Freiburg, 79106 Freiburg, Germany; 5 Department of Immunology, Genetics, and Pathology, Uppsala University, Uppsala, SE-751 85 Sweden; 6 Department of Physics, Technical University Munich, 85748 Garching, Germany

**Keywords:** Biochemistry, Prions, Protein folding, Cryoelectron microscopy

## Abstract

ATTR amyloidosis is one of the worldwide most abundant forms of systemic amyloidosis. The disease is caused by the misfolding of transthyretin protein and the formation of amyloid deposits at different sites within the body. Here, we present a 2.97 Å cryo electron microscopy structure of a fibril purified from the tissue of a patient with hereditary Val30Met ATTR amyloidosis. The fibril consists of a single protofilament that is formed from an N-terminal and a C-terminal fragment of transthyretin. Our structure provides insights into the mechanism of misfolding and implies the formation of an early fibril state from unfolded transthyretin molecules, which upon proteolysis converts into mature ATTR amyloid fibrils.

## Abstract (web summary)

Systemic amyloidosis of the ATTR is one of the most abundant forms of systemic amyloidosis and caused by misfolding of the circulating blood protein transthyretin (TTR). Here the authors present the cryo-EM structure of patient-derived Val30Met ATTR amyloid fibrils which reveals that the protofilament consists of an N-terminal and a C-terminal TTR fragment and discuss implications for the mechanism of misfolding.

## Introduction

Systemic amyloidosis of the ATTR (amyloid fibril protein derived from transthyretin) type is a potentially life-threatening disease that is caused by the misfolding of the circulating blood protein transthyretin (TTR)^1,2^. The disease arises from the extracellular deposition of amyloid fibrils in multiple organs. It represents one of the most abundant forms of systemic amyloidosis in many Western countries but shows geographical hotspots in Portugal, Brazil, Sweden and Japan^3,4^. ATTR amyloidosis can lead to diverse disease phenotypes with cardiomyopathy and polyneuropathy being particularly prominent^3,5^. Treatment includes liver transplantation to remove the main TTR-producing organ and, in cases of severe cardiomyopathy, heart transplantation^5^. The disease can be pharmacologically addressed with the drug tafamidis that is approved in many countries to antagonize the unfolding of native TTR^6^. Gene silencing approaches are under development to downregulate the biogenesis of the precursor protein^1,7^.

TTR is a naturally tetrameric protein with a very high β-sheet content^8,9^. One TTR chain consists of 127 amino acids and the native tetramer acts as a transporter of thyroxin hormone or retinol-binding protein in the blood^10^. Well over 120 mutational variants of TTR are known to give rise to hereditary ATTR amyloidosis with Val30Met being a particular important one^3,4^. Two main fibril morphologies have been found in ATTR amyloid deposits. Type A fibrils are relatively short and haphazardly arranged in ultrathin sections of amyloidotic tissue. They consist of a mixture of N-terminally truncated and full-length TTR^11,12^. Type B fibrils are more elongated and arranged into bundle-like assemblies that primarily consist of full-length TTR protein^11,12^. All organs of a patient show either type A or type B fibrils^13^, and the fibril type of a patient does not change over time^14^. Type A fibrils are more common and adopted by wild-type TTR and the vast majority of analysed mutational variants from hereditary amyloidosis^12^. Type B fibrils are formed from only few mutational variants of TTR, including Val30Met, which can give rise to either type A or type B fibrils in different patients. Interestingly, the fibril type correlates in Val30Met patients with the disease manifestation and the outcome of treatment^3^.

The mechanism by which TTR misfolds inside the body is unknown. Recombinant TTR was shown to form fibrils in vitro under slightly acidic conditions that destabilize the native state of the protein^15^. A reduced native-state stability was also described for several pathogenic TTR mutations, including Val30Met, suggesting that unfolding triggers fibril formation^16,17^. However, alternative fibrillation mechanisms have been proposed that do not require a major conformational rearrangement of the protein fold, describe an assembly of native-like TTR protomers or depend on the initial proteolytic cleavage of the precursor protein and the formation of a more amyloidogenic fragment^8,18–20^. In the absence of detail structural information on ATTR amyloid fibrils, it is difficult to further resolve these issues and to develop a fundamental molecular understanding of the mechanism of disease.

Previous analysis of negatively stained transmission electron microscopic (TEM) specimens of Val30Met ATTR amyloid fibrils revealed a square-like fibril cross-section, a hollow fibril core and the presence of four protofilaments^21^. X-ray diffraction demonstrated the amyloid-typical cross-β-sheet structure^22,23^ and led to a model consisting of four left-hand twisted cross-β-sheets^22^. Residue-specific structural information could be provided for fibrils grown in vitro from recombinant TTR protein^24^ or an 11-residue TTR fragment^25^ but it is unknown to what extent these fibrils correspond to the fibrils in ATTR patients. Taking advantage of a recently described protocol to purify fibrils from amyloidotic tissue^26^, we determined the structural characteristics of the amyloid fibrils from two patients with Val30Met ATTR amyloidosis. Cryo-electron microscopy (cryo-EM) yielded a structural reconstruction at 2.97 Å resolution, showing a single protofilament fibril with an asymmetrical cross-section.

## Results

### Primary structural heterogeneity of the fibril protein

ATTR amyloid fibrils were isolated from the hearts of two patients with type A Val30Met ATTR amyloid fibrils. Both patients show polyneuropathy with cardiac involvement. The used fibril extraction protocol was developed previously as particularly gentle methodology that avoids chemically or physically denaturing conditions and that does not disrupt the structure of the fibrils in the course of purification^26,27^. The extracted fibrils are relatively short; they show a mean width of 7.7 nm but lack well-resolved crossovers (Supplementary Fig. 1b, c). Their morphology matches closely to the structure of type A ATTR amyloid fibrils in the tissue prior to extraction^11^, indicating that the extraction protocol does not substantially alter the fibril morphology. Denaturing protein gel electrophoresis and Coomassie staining reveal a series of main fibril proteins with an apparent molecular mass of 7–12 kDa (Supplementary Fig. 1a). Full-length TTR is also visible in the fibril extracts by gel electrophoresis, although only at very low levels (Supplementary Fig. 1a). Reversed phase chromatography (RPC) shows similar chromatographic elution profiles with the fibril protein from both patients (Supplementary Fig. 2). The main protein components elute at 45–55% solvent B.

Mass spectrometry shows that the fibril proteins arise primarily from the C-terminus of TTR. Both patient samples contain molecular species corresponding to the masses of all TTR fragments from TTR(44–127) to TTR(53–127), as well as several other TTR fragments (Supplementary Table 1). These C-terminal fragments are consistent with the previously reported N-terminal truncation of type A TTR fibrils^11^ and demonstrate a conserved pattern of truncation in both patients (Supplementary Fig. 3a). Separation of fibril proteins with RPC and analysis of the individual fractions with mass spectrometry revealed a range of TTR fragments from the N-terminal 50 residues, encompassing the variant position 30 (Supplementary Fig. 3b, c). These fragments originate partly from TTR-V30M and wild-type protein (TTR-wt) (Supplementary Fig. 3b), consistent with previous observations that type A Val30Met ATTR amyloid fibrils contain fibril proteins derived from mutant and wild-type protein^14^.

### Cryo-EM reconstruction and modelling

**Fig. 1:**
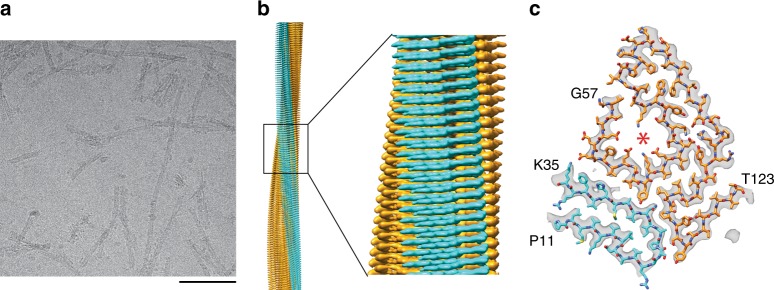
Cryo-EM reconstruction of the ATTR amyloid fibril. **a** Cryo-EM image of the extracted fibrils. Scale bar = 200 nm. **b** Side view of the reconstructed 3D map. N-terminal density: cyan; C-terminal density: orange. **c** Cross-sectional view of the reconstructed 3D map (grey), superimposed with a molecular model of the N-terminal (cyan) and C-terminal peptide segment (orange). Terminal amino acids are indicated in the figure. The internal cavity is marked with an asterisk

Cryo-frozen fibrils were imaged at 300 kV with a cryo-electron microscope (Fig. 1a). They are morphologically uniform under these conditions, although subtle variations in morphology or divergent fibril states of low abundance cannot be excluded. Two-dimensional (2D) class averages show a clear ~4.8 Å spacing (Supplementary Fig. 4a, b) arising from the fibril cross-β structure. A three-dimensional (3D) map reconstructed based on the cryo-EM images achieved a spatial resolution of 2.97 Å (Supplementary Table 2) based on the 0.143 Fourier shell correlation (FSC) criterion (Supplementary Fig. 4b). The fibril is polar, possesses C1 symmetry and consists of a single, twisted protofilament (Fig. 1b). The orientation of the twist could not be determined directly with platinum side shadowing and TEM, and the handedness was deduced from the fitting of a left-hand twisted 3D map or an inverted version hereof with a molecular model of TTR (Fig. 1b, c). This analysis revealed that only the left-hand twisted 3D map produced a good match to a polypeptide chain containing L-amino acids. We obtained a model resolution of 2.97 Å and a MolProbity score of 2.3 (Supplementary Table 3). All peptide bonds, including the five X-proline bonds, are present as *trans* isomers.

### Fold of the fibril protein

**Fig. 2:**
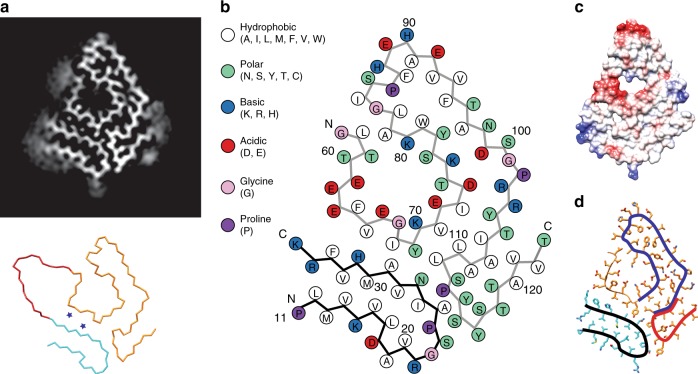
Packing and overall fold of the fibril protein. **a** Top: cross-sectional view of a 5-Å thick slice of the 3D map; bottom: Cα-trace of TTR residues Pro11-Lys35 (cyan) and Gly57-Thr123 (orange). Residues Ala36-His56 are modelled in an arbitrary conformation, showing the ability of this segment to connect the N- and C-terminal segment. The blue asterisks indicate the position of two density features not captured by our model. **b** Packing scheme of one cross-sectional layer. **c** Electrostatic surface profile of one molecular layer of the fibril. **d** The fibril protein contains arches at residues Pro11-Lys35 (black), Lys70-Leu111 (blue) and Thr106-Thr123 (red)

The fibril cross-section shows two continuous density regions presenting high structural order (Fig. 1c). The larger density segment fits with residues Gly57-Thr123 of TTR, the smaller density with residues Pro11-Lys35. These data show that the protein N-terminus constitutes an integral part of the fibril structure. Weak density occurs adjacent to these ordered regions, suggesting that residues Gly1-Cys10, Ala36-His56 and Asn124-Glu127 are structurally disordered or absent (Fig. 2a). The residue segment intermediate to the two ordered density regions (Ala36-His56) is large enough to readily fit in between the N- and C-terminal density of the fibril core (Fig. 2a), which implies that full-length TTR is able to fit one molecular layer of the fibril. As full-length TTR is present in these fibrils (Supplementary Fig. 1a), it is possible that the layers containing fragmented protein are interspersed by layers consisting of full-length TTR.

**Fig. 3:**
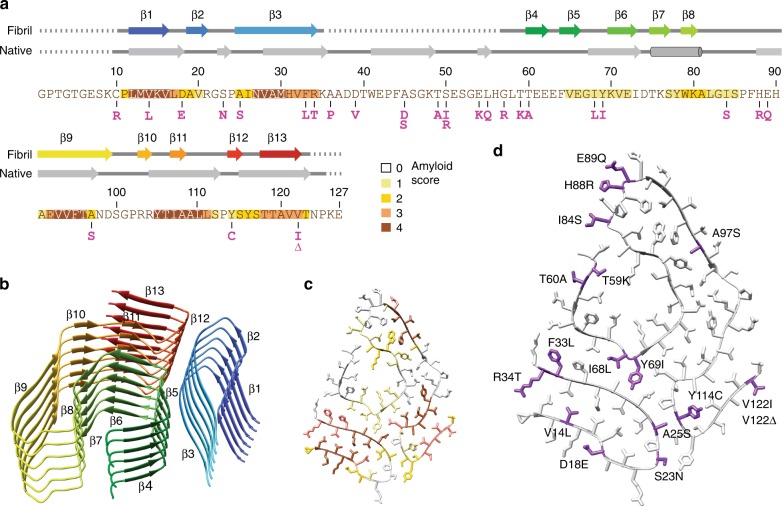
Location of the β-strand structure, mutational variants and aggregation-prone segments. **a** Amino acid sequence of Val30Met TTR and other mutational variants (magenta) known to give rise to type A fibrils. Background colour coding of the sequence shows the theoretic aggregation score (see **c**). Above the sequence are schematic representations of the secondary structure elements of native Val30Met TTR from protein data bank (PDB) entry 3DJT^56^ and of the fibril protein (PDB entry 6SDZ, this study). Arrows: β-strands; cylinders: α-helices; dotted line: residues not seen in the crystal structure or cryo-EM structure. **b** Ribbon diagram of a fibril stack showing six molecular layers. Rainbow colour from N- (blue) to C-terminus (red) as in **a**. **c** Location of the highly aggregation-prone segments according to the aggregation score 0–5 as indicated in **a**. **d** Schematic view of the fibril cross-section showing the position of the mutational variants of TTR that are known to form type A fibrils

The fibril cross-section shows a relatively compact protein fold that resembles the shape of a spearhead (Fig. 2). There is an internal cavity that is lined with polar and ionic residues and probably water-filled (Fig. 2b). Polar and charged amino acid residues define also the solvent-exposed surface on the outside of the fibril (Fig. 2b), similar to previously reported cross-β fibrils^28–30^. The fibril cross-section possesses acidic and basic patches (Fig. 2c) and consists of three arches that are formed by residues Pro11-Lys35, Lys70-Leu111 and Thr106-Thr123 (Fig. 2d). There are 13 β-strands (β1–β13) that extend between the residues Leu12-Val16, Ala19-Arg21, Ala25-Arg34, Thr60-Glu62, Phe64-Glu66, Lys70-Ile73, Thr75-Ser77, Trp79-Lys80, Ala91-Asp99, Arg103-Arg104, Ile107-Ala108, Tyr114-Ser115 and Thr118-Val122 (Fig. 3a). Strands β1–β3 occur within the N-terminal, strands β4–β13 within the C-terminal segment (Fig. 3a). Stacking up the fibril proteins along the fibril *z*-axis generates the cross-β-sheets. All cross-β-sheets have parallel strand–strand contacts (Fig. 3b).

Analysis of the sequence of TTR-V30M with computational tools^31–35^ reveals several highly amyloidogenic segments within the fibril core, while residues Gly1-Cys10 and Ala36-His56, which do not participate in forming the fibril core (see above), lack highly aggregation prone segments (Fig. 3a). Each of the three arches contains at least two aggregation-prone segments that are paired in the 3D structure (Fig. 3c). Appropriately docking the N-terminal arch onto the C-terminal segment produces non-local interactions (Supplementary Fig. 5a) and establishes the fibril protein fold. Nineteen of 29 (66%) known mutational variants of TTR that form type A amyloid fibrils^12^ affect a residue within the fibril core (Fig. 3d). This percentage value relates closely to the percentage of TTR residues within the fibril core (92 out of the 127 residues or 72%), indicating that there is no significant preference of the mutations for a core position.

### Molecular interactions within fibril structure

The interactions that extend within the cross-sectional plane are defined mainly by the amino acid side chains. In the N-terminal arch, an extensive hydrophobic core is formed by two leucine, one proline, one isoleucine and five valine residues as well as the variant position 30 (Supplementary Fig. 5b). Our 3D map is consistent with either a methionine or a valine at position 30, corresponding to the observation of TTR-wt and TTR-V30M-derived fibril proteins by mass spectrometry (Supplementary Fig. 3b, c). The two arches of the C-terminal fragment encompass hydrophobic, polar and ionic interactions (Fig. 2c), such as a cluster of buried salt bridges at residues Asp74, Lys76, Asp99 and Arg103 (Supplementary Fig. 5c). The interface between the N-terminal arch and the C-terminal segment involves a network of non-local interactions (Supplementary Fig. 5a) and contains two density features that are not captured by our model (Fig. 2a). However, it is currently unclear whether they represent molecular inclusions or artefacts of the image processing.

The interactions in the direction of the main fibril axis include, first of all, the intermolecular backbone–backbone hydrogen bonds of the fibril cross-β-sheets (Supplementary Fig. 6a). In addition, there are extensive side chain–side chain contacts along the fibril *z*-axis, for instance, between stacked aromatic side chains (Supplementary Fig. 6b). The polypeptide chains show a notable height change of 5.1 Å within N-terminal segment (Supplementary Fig. 6c) and of 6.4 Å within the C-terminal segment (Supplementary Fig. 6d). This height change sterically interdigitates the 4.8-Å spaced layers of the fibril, for example by the formation of cross-layer salt bridges (Supplementary Fig. 6e), conferring mechanical resistance and producing different tip structures at either fibril end.

### The fibril protein fold differs substantially from native TTR

**Fig. 4:**
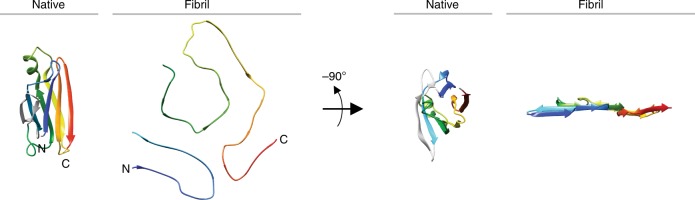
Comparison of the fibril protein with a natively folded TTR protomer. Pairwise arrangement of ribbon diagrams of one natively folded Val30Met TTR protomer (PDB entry 3DJT)^56^ and of the fibril protein. Both structures are correspondingly rainbow coloured from N- (blue) to C-terminus (red). Light grey segments in the native structure are disordered in the fibril

The fibril protein fold differs profoundly from the native conformation of TTR-Val30Met, which contains nine β-strands per protomer along with a single α-helix (Fig. 3a). The β-sheets within the native state show many antiparallel strand–strand interactions (Fig. 4), while the fibril contains exclusively parallel strand–strand interactions (Fig. 3b). The positions of the β-strands in the fibril protein sequence also does not correlate well with the position of the β-strands in native TTR (Fig. 3a). Tetrameric, functional TTR consists of well-folded, globular protomers, while the ATTR fibrils contain relatively flat fibril proteins (Fig. 4).

## Discussion

In this research, we determined the cryo-EM structure of a type A ATTR amyloid fibril, the most common fibril morphology in systemic ATTR amyloidosis^12^. The fibril protein presents a relatively flat, β-sheet-rich conformation that encloses an internal cavity (Fig. 1c). The fibril protein fold differs substantially from the globular fold of native TTR-V30M protein (Fig. 4). We can identify two TTR fragments, a C-terminal fragment (residues Gly57-Thr123) that corresponds to the previously reported ATTR fibril proteins^11^ and an N-terminal fragment encompassing the residues Pro11-Lys35. Therefore, the analysed type A ATTR amyloid fibril is mixed in the sense that it contains two non-homologous polypeptide chains. Taken together with the recently reported cryo-EM structures of amyloid fibrils from systemic AA and AL amyloidosis^36–38^, detail structural information is now available for the three classical types of systemic amyloidosis.

**Fig. 5:**
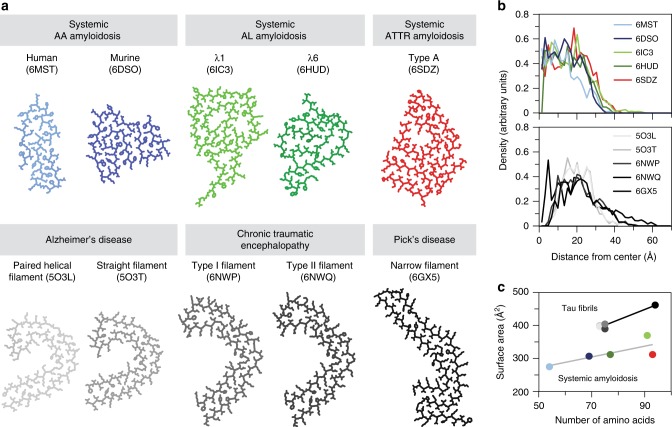
Comparison of amyloid fibrils from systemic amyloidosis with tau-derived fibrils. **a** Views of the cross-sectional layers of five fibrils from systemic AA^36^, AL^37,38^ and ATTR amyloidosis and tau-derived fibrils from Alzheimer’s^39^, chronic traumatic encephalopathy^41^ and Pick’s disease^40^. **b** Radial mass distributions of one protein stack. **c** Surface area of one protein stack plotted against the number of amino acids in the fibril core. A linear fit was added to guide the eye. The colour coding is kept consistent in all panels. Source data are provided as a Source Data file

These structures have in common that the fibril cross-β-sheets possess parallel strand–strand contacts and that the fibril proteins adopt, despite internal cavities, relatively compact protein conformations (Fig. 5a, b). They differ with respect to this feature from tau protein fibrils, which show a relatively elongated protomer conformation and consist of mainly two laminated cross-β-sheets (Fig. 5a, b). The greater compactness of the typically extracellular fibrils from systemic amyloidosis compared with the typically intracellular tau fibrils is also reflected in their smaller surface area when normalized to the number of amino acid residues (Fig. 5c). Furthermore, fibrils in systemic amyloidosis are usually dominated by a single polymorph^36–38^, while tau fibrils typically present two or more abundant fibril morphologies per patient^39–41^.

Another difference of fibrils in systemic AA, AL and ATTR amyloidosis from tau fibrils is that they arise from precursor proteins that can normally fold into compact globular conformations. The fibril precursor in systemic AA amyloidosis is serum amyloid A (SAA), an all-α protein that shows a native conformation that is radically different from the all-β fold of AA amyloid fibrils^36^. The fibril precursors in systemic AL and ATTR amyloidosis (immunoglobulin light chain and TTR) are β-sheet rich. However, even for AL and ATTR fibril protein conformations it was found that these differ from the native conformations of the respective precursor proteins and that they present substantially rearranged sets of intramolecular interactions. Hence, it is clear that the fibrils in all three forms of amyloidosis form from relatively unfolded polypeptide chains. And indeed, Val30Met and several other mutations in hereditary ATTR amyloidosis were found to destabilize the native state of TTR^16,17^.

Wild-type TTR and 28 previously variants of TTR from hereditary ATTR amyloidosis produce type A amyloid fibrils^12^. Ten mutations (Cys10Arg, Ala36Pro, Asp39Val, Ala45Asp, Ala45Ser, Thr49Ala, Ser50Ile, Ser50Arg, Glu54Lys, Leu55Gln) do not affect the fibril core and occur within the disordered segments (Fig. 3a). Ten substitutions exchange a surface residue of the fibril protein (Asp18Glu, Ser23Asn, Arg34Thr, Gly57Arg, Thr60Ala, Ile84Ser, His88Arg, Glu89Gln, Val122Thr, Val122Δ). One mutation (Thr59Lys) replaces a residue that protrudes into the water-filled cavity, leading to a better charge balance of the residues lining the cavity. Eight mutations (Val14Leu, Ala25Ser, Val30Met, Phe33Leu, Ile68Leu, Tyr69Ile, Ala97Ser, Tyr114Cys) concern positions that are not solvent exposed but the substitutions are rather conservative. Although it is difficult to estimate the effect of mutations on the fibril structure, the majority of mutations cannot be associated with any obviously disturbing or stabilizing effects on the fibril structure and may rather act on the fibril precursor or on aggregation pathway. Consistent with this notion, there is no discernible preference for mutations to occur in the fibril core (see above) and many previous studies suggest TTR unfolding to be crucial for fibril formation^6,15–17^.

Our study has two main implications for the mechanism of misfolding. The first implication is that the native conformation of TTR must be largely unfolded to facilitate the formation of fibrils inside the body (see above). The second implication concerns the proteolytic fragmentation of the precursor protein, which represents a common feature of fibrils in systemic ATTR, AA and AL amyloidosis^11,36,37^. In all three diseases, it is a major unresolved question whether proteolysis follows fibril formation or vice versa, and only for few cases the order of events could be established with good confidence. One such case is the N-terminal truncation of SAA protein in the common variant of systemic AA amyloidosis. A recent cryo-EM structure revealed the truncated N-terminus to be buried within the fibril core where it is unable to accommodate additional N-terminal residues and where it is inaccessible to proteases^36^. While these data provided strong evidence that proteolysis precedes fibril formation, the order of events remains unclear in most other cases, including the formation of type A ATTR amyloid fibrils.

**Fig. 6:**
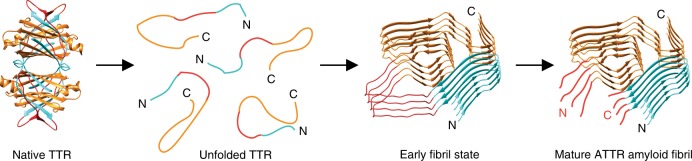
Possible mechanism of misfolding of TTR protein. The first step is the disassembly and unfolding of the native tetramer, followed by the assembly of the polypeptide chains into an early fibril state. The last step is the proteolytic cleavage of TTR in the structurally disordered segment of residues Ala36-His56 (red) and the formation of the mature ATTR amyloid fibril. The N- and C-terminal segments of the fibril are colour coded orange and cyan, respectively

The co-existence of an N- and a C-terminal TTR segment in one fibril, which are revealed here (Fig. 1), and the relative spatial arrangement of the two segments, which is compatible with full-length TTR (Fig. 2a) that is also present in the fibril (Supplementary Fig. 1a), argue that fibril formation precedes proteolysis. Alternatively, a complex and rather unlikely mechanism would have to be assumed in which full-length TTR is initially cleaved into two unfolded polypeptide chains that, instead of being degraded, co-assemble at 1:1 molar ratio into a fibril structure that adopts an arrangement compatible with full-length TTR. The much more straightforward and likely mechanism arising from our data is instead the initial disassembly and unfolding of the TTR tetramer (Fig. 6), which is followed by the association of unfolded polypeptide chains into an early fibril state. This early fibril state consists of full-length TTR and contains the low amyloidogenic segment at residues Ala36-His56 in a solvent-exposed conformation (Fig. 3a). Proteolytic cleavage of the early fibril state within this segment ultimately generates the mature ATTR amyloid fibrils and leads to N-terminally truncated TTR fragments described in earlier studies^11,12^. In fact, several studies report the observation of N-terminal peptide fragments of TTR, such as the fragment Val16-Arg34, in tryptic digests of type A ATTR amyloid fibrils^11^. However, since full-length TTR was present in the fibril extracts, it remained unclear whether the peptide fragment was a part of the fibril before trypsin was added.

Given that the structure of an in vitro formed TTR fibril has native-like structural characteristics^24^ and differs in this property starkly from the presently analysed fibril, our data underscore the importance of investigating patient-derived amyloid fibrils when analysing the molecular basis of amyloid diseases. This conclusion is further substantiated by evidence coming from other fibril proteins that show the cross-β fibrils formed in vivo differ from the reported fibrils formed by corresponding polypeptide chains in vitro^27,42^. One possible way to rationalize these differences is to assume that fibril formation in vivo involves one or several molecular components that are lacking in the respective in vitro samples and thus induce a different fibril structure. Support for this view is provided by observations of molecular inclusions existing in previously reported ex vivo fibrils^37,41^ and of weak density features that decorate many ex vivo cross-β fibrils^36–41^. While the molecular identity of these components remains to be established in most cases, it is reasonable to assume that they could modify the assembly pathway. If so, preventing their association with pathogenic polypeptide chains could represent a possible strategy to prevent the formation of disease-relevant amyloid fibril morphologies.

## Methods

### Source of ATTR fibrils

Amyloid fibrils were extracted from the left ventricle of the heart of two patients with hereditary ATTR amyloidosis. Patient I (internal reference number POW 116) was a 78-year-old man, who had a progressive symmetrical polyneuropathy starting in lower legs, gastrointestinal problems with malabsorption and a severe progressive restrictive cardiomyopathy ending in cardiac failure. In addition, he had pacemaker due to atrial fibrillation with slow ventricular rate. He died from cardiac insufficiency in 1979. At autopsy, severe cardiomegaly was found (heart weight 835 g). ATTR nature of the amyloid was established in 1987. Heterozygosity for Val30Met is demonstrated with this study. Patient II (internal reference number POW 237) was a 75-year-old man heterozygous for the Val30Met TTR mutation and died after >10 years with ATTR amyloidosis, manifesting as progressive polyneuropathy and cardiac insufficiency (patient number 7 from previous study^43^). His cardiac ATTR was analysed earlier and shown to be type A and to contain both mutated and wild-type TTR^11^. The material collection and use was performed under a valid permit by the Ethical Committee at Uppsala University Hospital (Ups 01-083).

### Fibril extraction from the tissue

The extraction of fibrils from human tissue is based on the water extraction protocol from Pras et al.^44^ but modified according to Annamalai et al.^26^. In brief, ~250 mg of frozen human tissue per patient were thawed at room temperature and diced into small pieces with a scalpel. The diced tissue was transferred to a reaction tube and washed with 1 ml Tris-calcium buffer (20 mm Tris, 138 mm NaCl, 2 mm CaCl_2_, 0.1 % NaN_3_, pH 8.0). The suspension was centrifuged for 5 min at 3100 × *g* and 4 °C. The supernatant was collected and the Tris-calcium buffer washing step was repeated four more times. After the fifth washing step, the pellet was gently resuspended in freshly prepared collagenase solution and incubated overnight on a reciprocating shaker set to 500 rpm at 37 °C. Then the suspension was centrifuged for 30 min at 3100 × *g* and 4 °C. The supernatant was collected and the pellet was gently resuspended in 1 ml Tris–ethylenediaminetetraacetic acid (EDTA) buffer (20 mm Tris, 140 mm NaCl, 10 mm EDTA, 0.1 % NaN_3_, pH 8.0). The suspension was centrifuged for 5 min at 3100 × *g* and 4 °C. The supernatant was collected and the washing step with Tris–EDTA was repeated nine more times. After the tenth washing step, for the extraction of amyloid fibrils, the pellet was gently resuspended in 500 µl ice-cold water. The suspension was centrifuged for 5 min at 3100 × *g* and 4 °C. Eventually, the amyloid fibril-containing supernatant was collected and the extraction step was repeated nine more times. The material from the two patients was never combined and always analysed separately. The biochemical analysis was approved by the ethical committees of Ulm University (210/13).

### RPC purification of proteins from the extracted fibrils

The water extracts were pooled and disaggregated in 6 M guanidine hydrochloride, 20 mM sodium phosphate buffer, pH 6.5. The sample was incubated overnight on a reciprocating shaker set to 200 rpm. Subsequently, 0.2% (v/v) trifluoroacetic acid (TFA) was added to the sample. Then the sample was loaded on a 3-ml Resource RPC column, which was equilibrated with RPC solution A (0.1% (v/v) TFA). After loading the sample on the column, which was washed with RPC solution A, the elution was started by applying a linear gradient from 0% to 100% RPC solution B (0.1% (v/v) TFA in 86% (v/v) acetonitrile) over 35 column volumes. The eluted absorbance signals at 215 and 280 nm were recorded to detect peptide bonds and aromatic amino acids, respectively. Fractions of 3 ml were collected, of which 200 µl were taken and concentrated by a factor of 10 for gel electrophoretic analysis. Collected fractions were lyophilized for 48 h and stored at −80 °C.

### Denaturing protein gel electrophoresis

Twenty microlitres of the sample were mixed with 2.9 µl NuPAGE Sample Reducing Agent (10×) (Thermo Fisher Scientific) and 6.7 µl 4× concentrated NuPAGE LDS Sample Buffer (Thermo Fisher Scientific) via vortexing. The mixture was incubated on a block heater at 95 °C for 10 min. Six microlitres of protein marker and 10 µl of the sample were loaded on a NuPAGE 10–12% Bis-Tris LDS-PAGE gel (Thermo Fisher Scientific), respectively. Five microliters of NuPAGE Antioxidant was added to 1× concentrated NuPAGE MES SDS Running Buffer. Electrophoresis was performed at 180 V for 35 min at room temperature. The gel was stained with Coomassie staining (0.25% (w/v) Coomassie Brilliant Blue G-20, 20% (v/v) ethanol, 10% (v/v) acetic acid) solution for 1 h and overnight in 20% (v/v) acetic acid and 10% (v/v) ethanol.

### Mass spectrometry

Sample preparation for mass spectrometry was performed as described previously^45^. In brief, samples of the extracted human ATTR amyloid fibrils were denatured in 6 M guanidine hydrochloride and desalted using ZipTips C18 (Merck Millipore). Following carbamidomethylation, the protein was applied on a monolithic ProSwift (250 mm × 1 mm I.D.) reversed phase column and eluting fragments were measured using an Orbitrap Velos Pro (Thermo Scientific) mass spectrometer. For identification of high-molecular-weight TTR fragments, averaged mass spectra were deconvoluted. Subsequently, the resulting deconvoluted monoisotopic mass lists were processed using in-house developed Excel VBA (Microsoft Corporation) scripts to calculate deconvoluted mass spectra and assignment of proteolytic fragments to specific masses. For identification of peptide-length fragments, PEAKS Studio X (BSI, Waterloo, Canada) was employed with an unspecific digest, oxidation of methionine and carbamidomethylation of cysteine as variable modifications and further default parameters for correlation of mass spectra with both TTR-wt and TTR-V30M sequences.

### Negative-stain TEM

Negative-stain TEM specimens were prepared by loading 4 µl of the sample onto a formvar and carbon-coated 200 mesh copper grid (Electron Microscopy Sciences). After incubation of the sample for 1 min at room temperature, the excess solvent was removed with filter paper. The grid was washed three times with water and stained three times with 2% uranyl acetate solution. Grids were examined in a JEM-1400 transmission electron microscope (JEOL) that was operated at 120 kV. The quantification of the fibril width was performed using the program GIMP.

### Cryo-TEM

The amyloid fibril-containing water extract was concentrated by a factor of 1.5 and centrifuged at 5511 × *g* for 1 h. A 3-μl aliquot was applied to a glow-discharged holey carbon-coated grid (C-flat 1.2/1.3 400 mesh from Electron Microscopy Sciences), blotted with filter paper after an incubation time of 8 s (temperature 21°, humidity >90%) and plunge-frozen in liquid ethane using a Vitrobot Mark 3 (Thermo Fisher Scientific). Grids were screened using a JEM-2100 transmission electron microscope (Jeol) at 200 kV. For image acquisition, a K2-Summit detector (Gatan) in counting mode on a Titan Krios transmission electron microscope (Thermo Fisher Scientific) at 300 kV was used with a Gatan imaging filter with a 20 eV slit. Supplementary Table 2 lists the data acquisition parameters.

### Helical reconstruction

The raw data movie frames were gain-corrected with IMOD^46^ and aligned, motion-corrected and dose-weighted using MOTIONCOR2^47^. Gctf^48^ was used to estimate the contrast transfer function from the aligned and motion-corrected images. RELION 2.1^49^ was used for the helical reconstruction of the fibril density. Fibrils were selected manually from the aligned, motion-corrected micrographs. Segments were extracted with a box size of ~200 Å and an inter-box distance of ~7% of the box length. Approximately 100k particles were extracted. Reference-free 2D classification with a regularization value of *T* = 2 produced class averages showing the helical repeat along the fibril axis. Class averages were selected on how well the helical repeat along the fibril axis was visible. From the selected class averages, 200 randomly picked particles per class were used to generate an initial 3D model de novo using the stochastic gradient descent algorithm implemented in RELION. This initial model was low-pass filtered to 60 Å for 3D classification with *K* = 4. Particles from three out of four classes with a similar well-defined structure were selected. The ~70k particles from these three classes were then used for a second 3D classification run with *T* = 10 using as a reference the model showing most features from the previous classification. This classification produced a model showing a clear continuous peptide backbone in the cross-section. The *T*-value was increased to 20 for a third 3D classification run, that yielded a model showing, additionally to the β-strand separation, side-chain densities. Auto-refinement and post-processing with a soft-edged mask and an estimated map sharpening *B*-factor of −50 Å² yielded the final model with a twist of −1.19° and 4.825 Å. 3D classification and auto-refine processes used a central part of 10% of the intermediate reconstruction^49^. Resolution estimates (2.97 Å) were obtained from the FSC at 0.143 between two independently refined half-maps.

### Model building and refinement

An initial model was built manually into a *B*-factor sharpened map in Coot^50^. Using the initial model, the central well-resolved section of the map was masked and used for further refinement. A locally *B*-sharpened map generated with LocScale^51^ assisted model building. The resulting model was refined at first by real space refinement using phenix.real_space_refine^52^ with NCS constraints and secondary structure restraints and finally with refmac5 in the CCPEM suite^53^ using NCS constraints. The model quality was assessed using Molprobity^54^.

### Prediction of aggregation-prone regions

The amyloid score for each residue was determined on the basis of the residue-specific prediction of amyloid-prone regions with the programs WALTZ^31^, TANGO^32^, AmylPred^33^, Foldamyloid^34^ and Aggrescan^35^. An amyloid score of 0 implies that none of the programs identified this residue as aggregation prone, while an amyloid score of 5 means that all five programs suggest that this residue promotes amyloid formation. The following threshold values were used: WALTZ, >0.00; TANGO, beta sheet aggregation >0.00; Fold amyloid, >21.4 for five consecutive residues; Aggrescan, >−0.02; AmylPred, sequence regions determined as hits by the Consensus method.

### Surface area calculation on the lateral surface of fibrils

Fibril segments consisting of at least seven peptide layers with the fibril axis aligned along the *z*-axis were used for calculating the surface area per unit length (1 Å) along the *z*-axis. A surface probe with radius 2.5 Å was first used to mark atoms on the lateral surface excluding cavities in the fibril interior and accessible areas on the top or bottom segment of the fibril segment. Larger cavities still accessible by a 2.5 Å probe were identified manually and excluded from the surface area calculation. The Shrake and Rupley method^55^ with a standard water probe radius of 1.4 Å was then employed to calculate the surface area of only the lateral fibril surface and to calculate the surface area per 1 Å in *z*-direction. In cases of fibrils with two protein stacks, surface area values represent averages over both.

### Radial atom density distribution

The centre of the central peptide layer of fibril segments with seven peptide layers was used as reference for radial atom density calculations. The atom density was calculated by counting the number of atoms within radial segments of 3.4 Å, which corresponds to the diameter of a carbon atom, at a given distance *r* from the centre along a segment in *z*-direction of 20 Å (+/−10 Å with respect to the centre of the fibril). The distance step size was 1.7 Å. The atom density was calculated after division of the number of atoms by the volume of the radial segment.

### Reporting summary

Further information on research design is available in the Nature Research Reporting Summary linked to this article.

## Supplementary information

Supplementary Information

Reporting Summary

Source Data

## Data Availability

The reconstructed cryo-EM map was deposited in the Electron Microscopy Data Bank with the accession code EMD-10150. The coordinates of the fitted atomic model were deposited in the Protein Data Bank under the accession code 6SDZ. The data that support the findings of this study are available from the corresponding authors upon reasonable request. The source data underlying Fig. 5b, c and Supplementary Figs. 1a, c, 2b, 3a, c and 4c are provided as a Source Data file.
