# Supplementary material for: Cryo-EM structure of a transthyretin-derived amyloid fibril from a patient with hereditary ATTR amyloidosis
Source: Nat Commun. 2019 Nov 1;10:5008. doi: 10.1038/s41467-019-13038-z (PMC6825171; doi:10.1038/s41467-019-13038-z)
Supplement: Supplementary file 1 — Supplementary Information [file 41467_2019_13038_MOESM1_ESM.pdf]

Supplementary Information

Cryo-EM structure of a transthyretin-derived amyloid fibril from a patient with hereditary ATTR amyloidosis

M. Schmidt et al.

Supplementary Figure 1

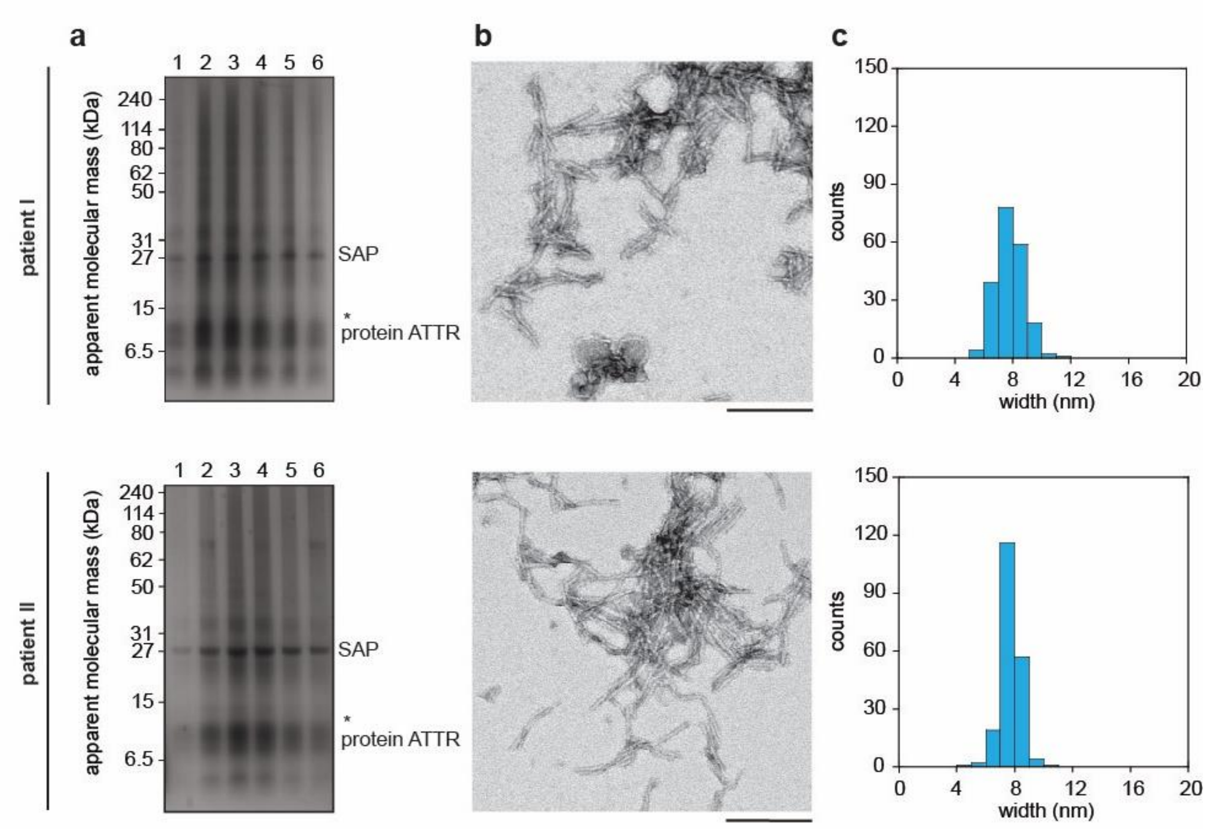

Supplementary Figure 1

Extraction of the amyloid fibrils from patient tissue.

Top row: patient I; bottom row: patient II. (a) Representative denaturing protein gels of the first 6 water extract fractions after Coomassie staining. The main fibril protein ATTR bands occur at an apparent molecular mass of 7-12 kDa. A weak band corresponding to full-length TTR is marked with an asterisk. SAP: serum amyloid P component. (b) TEM images of negatively stained fibril extracts. Scale bar = 200 nm. (c) Quantification of the fibril width based on negative stain TEM for fibrils (n = 200). The mean values ± standard deviation are 7.7 ± 0.9 nm for patient I and 7.7 ± 0.7 nm for patient II, respectively. Source data are provided as a Source Data file for (a) and (c).

Supplementary Figure 2

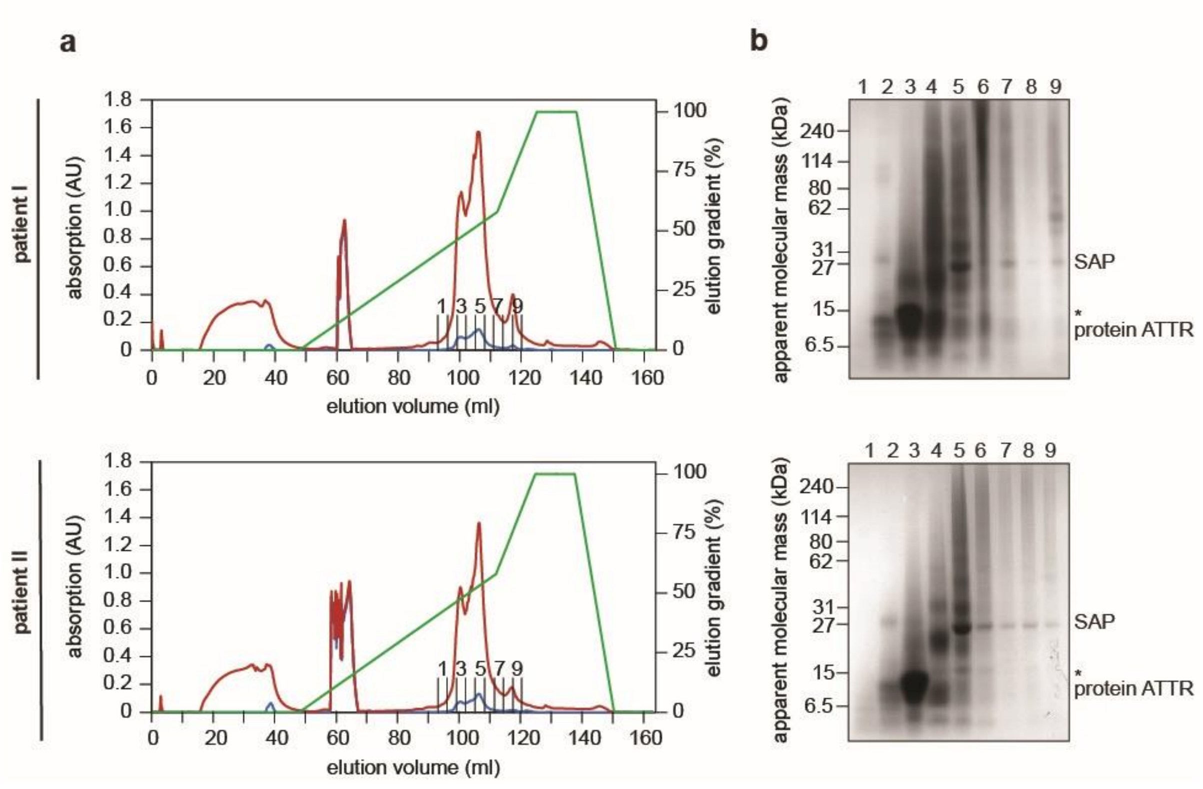

Supplementary Figure 2

Chromatographic purification of the fibril protein.

Top row: patient I; bottom row: patient II. (a) RPC chromatographic profiles of the fibril extracts of patients I and II. Absorption at 215 nm (red) and 280 nm (blue). Position of fractions 1-9 in the elution profile indicated. Concentration of solvent B in % (green). (b) Coomassie-stained denaturing protein gel of the fractions indicated in (a). Full-length TTR is marked with an asterisk. SAP: serum amyloid P component. Source data are provided as a Source Data file for (b).

Supplementary Figure 3

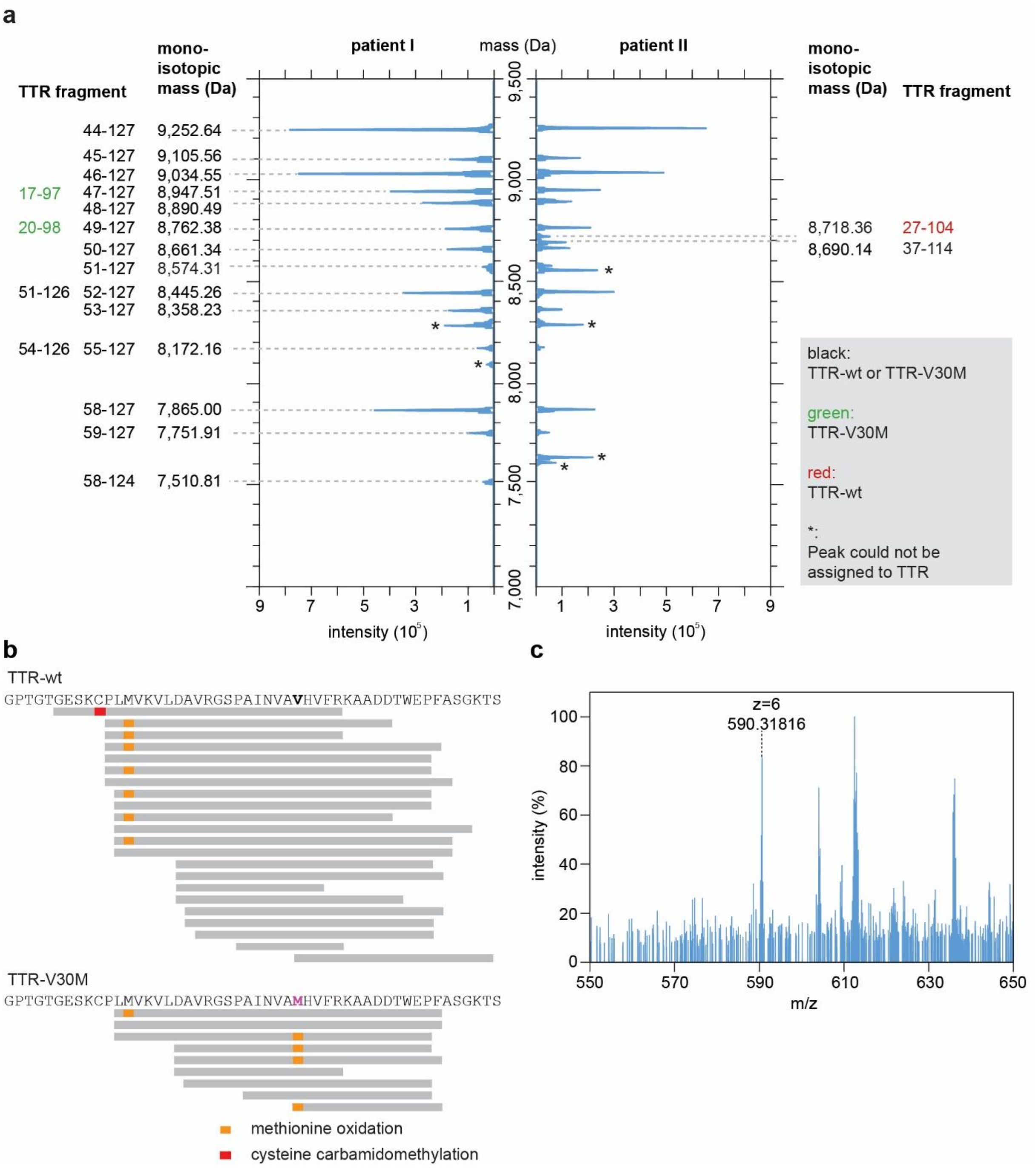

Supplementary Figure 3

Mass spectrometric analysis of the fibril extracts.

(a) Left: Deconvoluted mass spectra (spectral range 7,000-9,500 Da shown) of unfractionated fibril extracts from patients I and II. Black: fragment of TTR-wt or TTR-V30M, green: TTR-V30M fragment; red: TTR-wt fragment; *: monoisotopic mass could not be assigned within error (0.1 Da) to unmodified

fragments of TTR-wt or TTR-V30M protein (Table S1). (b) N-terminal fragments of TTR-wt (upper) and TTR-V30M (lower) in patient I fibril extracts detected by mass spectrometry after reversed phase separation. We considered Met oxidation (+ 15.99 Da, orange) and Cys carbamidomethylation (+ 57.02 Da, red) as possible modifications. Only fragments encompassing residue 30 shown. (c) Representative mass spectrum (spectral range m/z 550 to 650) showing the N-terminal fragment with sequence LMVKVLDAVRGSPAINVAMHVFRKAADDTWEP (residues 12-43, for z=6 theoretic m/z is 590.3154 ±0.0037) of TTR-V30M at an m/z value of 590.31816. Source data are provided as a Source Data file for (a) and (c).

Supplementary Figure 4

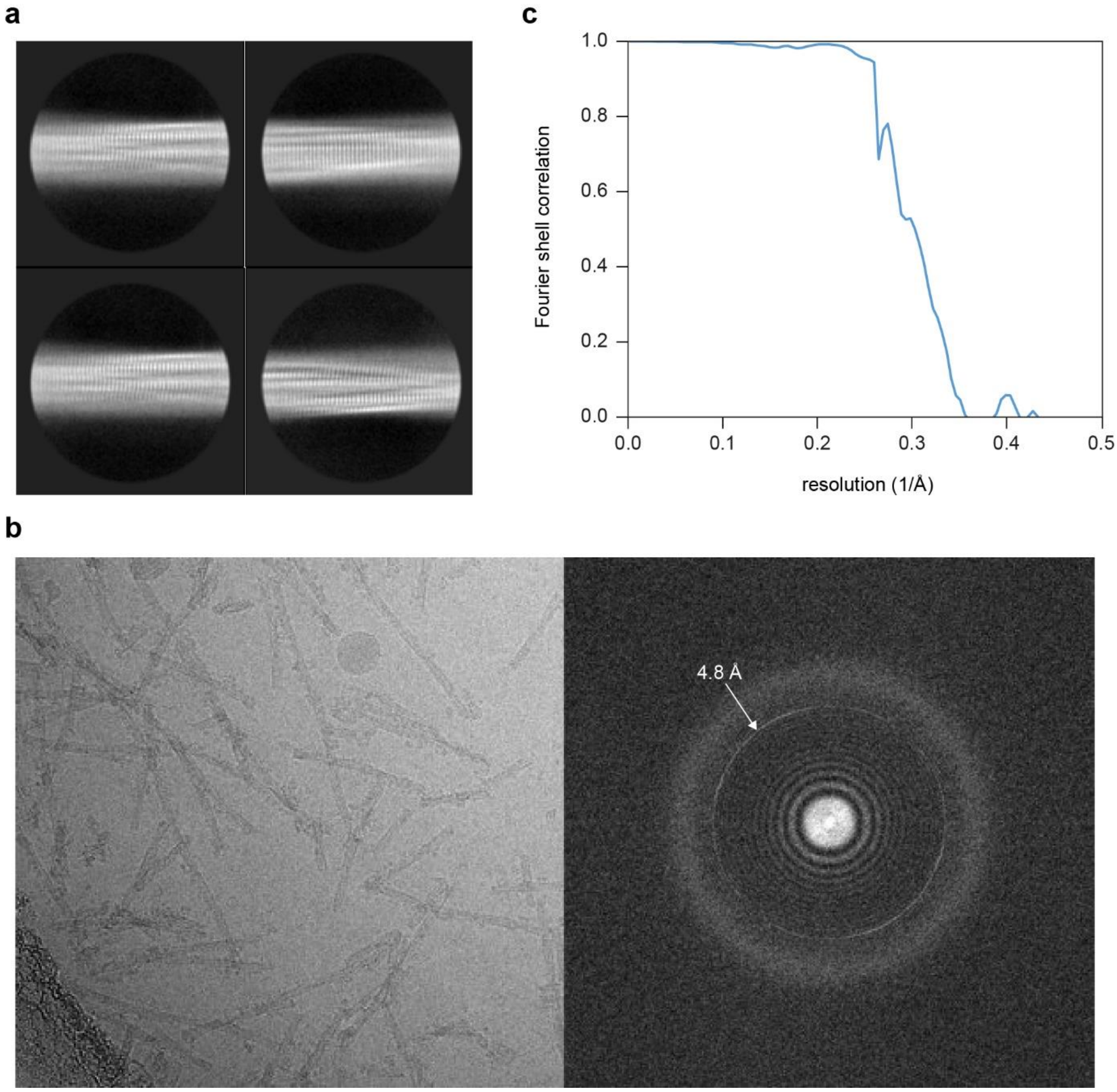

Supplementary Figure 4

Cryo-EM image processing characteristics.

(a) Representative 2D class averages. (b) Raw cryo-image of TTR fibrils (left), its Power spectrum with showing ~4.8Å reflection of β-sheets distance along the fibril axis (right). (c) FSC of two half maps. Source data are provided as a Source Data file for (b).

Supplementary Figure 5

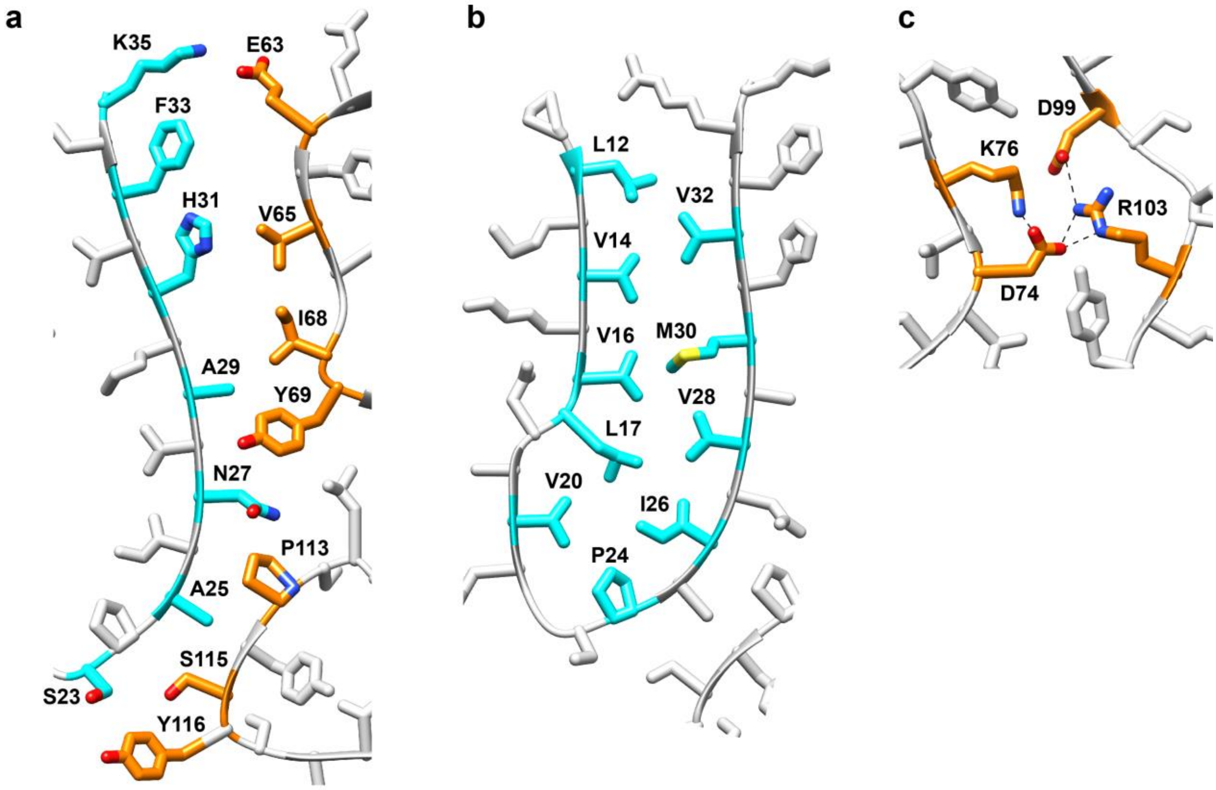

Supplementary Figure 5

Detail features of the fibril cross-section.

(a) Residues at the interface between the N-terminal (cyan) and the C-terminal segment (orange). (b) Cross-sectional view of the N-terminal segment with the residues forming the hydrophobic core coloured in cyan. (c) Buried salt bridges (dashed lines) formed by residues Asp74, Lys76, Asp99 and Arg103 within the C-terminal segment.

Supplementary Figure 6

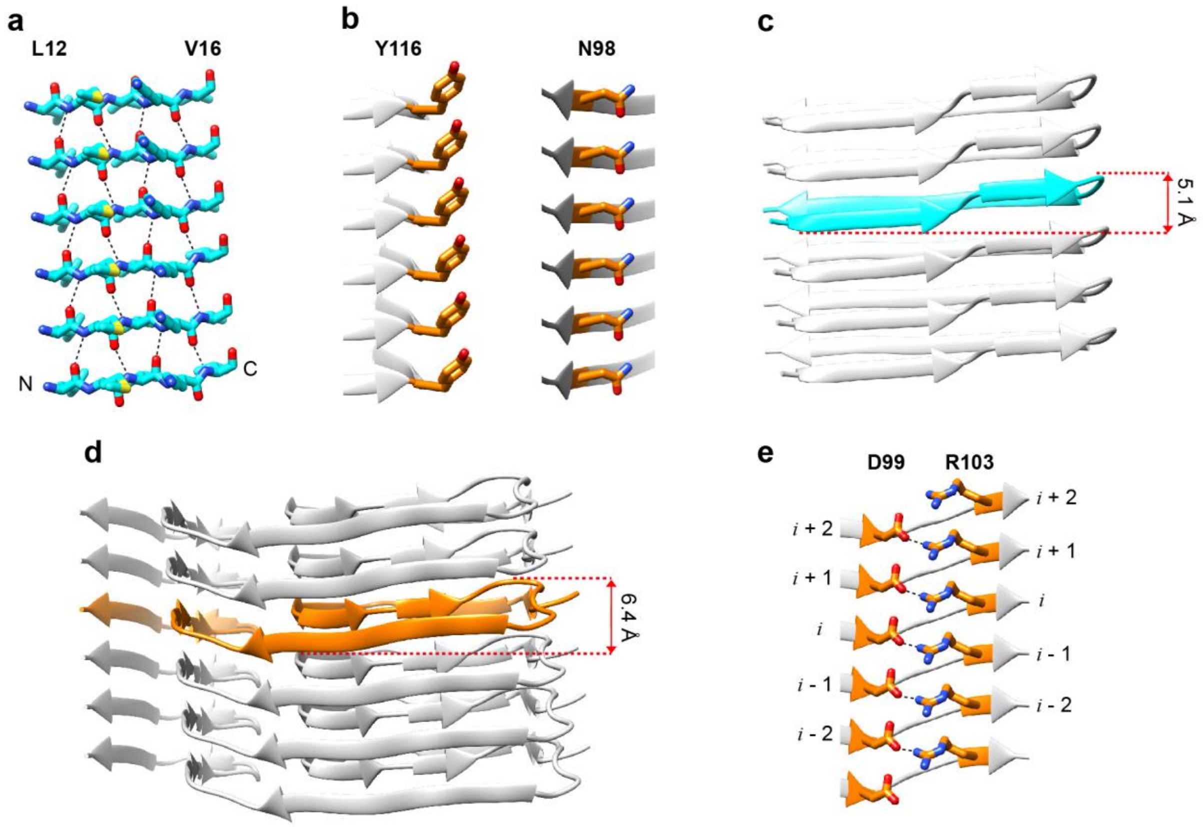

Supplementary Figure 6

Detail structural features along the fibril main axis.

(a) Hydrogen bond network within β-sheet 1, showing parallel interactions between the strands. (b) Stacking of aromatic (Tyr116) and polar (Asn98) residues along the main fibril axis. (c) Height change of the N-terminal segment. (d) Height change of the C-terminal segment. (e) Side chain interactions of Asp99 and Arg103 of different layers due to height differences in the C-terminal segment. The layer numbers are referred to as *i*, *i* + 1 etc.

Supplementary Tables

Supplementary Table 1

Experimental monoisotopic masses of patient I and II fibril extracts at 7,000-9,500 Da.

**Experimental mass (Da)**	**POW 116**	**POW 237**	**TTR-V30M**	**TTR-wt**	**Fragment/(Da)**
7,510.81	yes	-	yes	yes	58-124 (7,510.76)
7,751.91	yes	yes	yes	yes	59-127 (7,751.86)
7,865.00	yes	yes	yes	yes	58-127 (7,864.95)
8,172.16	yes	yes	yes	yes	54-126 (8,172.11)
	yes	yes	yes	yes	55-127 (8,172.11)
8,358.23	yes	yes	yes	yes	53-127 (8,358.18)
8,445.26	yes	yes	yes	yes	51-126 (8,445.21) #
			yes	yes	52-127 (8,445.21) #
8,574.31	yes	yes	yes	yes	51-127 (8,574.25)
8,661.34	yes	yes	yes	yes	50-127 (8,661.28)
8,690.14	-	yes	yes	yes	37-114 (8,690.23)
8,718.36	-	yes	-	yes	27-104 (8,718.28)
8,762.38	yes	yes	yes	-	20-98 (8,762.31) #
			yes	yes	49-127 (8,762.33) #
8,890.49	yes	yes	yes	yes	48-127 (8,890.42)
8,947.51	yes	yes	yes	-	17-97 (8,947.42) #
			yes	yes	47-127 (8,947.45) #
9,034.54	yes	yes	yes	yes	46-127 (9,034.48)
9,105.56	yes	yes	yes	yes	45-127 (9,105.52)
9,252.64	yes	yes	yes	yes	44-127 (9,252.58)

The table refers to the deconvoluted mass spectra in Supplementary Figure 3a. #: alternative assignments possible within error (0.1 Da).

Supplementary Table 2

Statistics of cryo-EM data collection and image processing.

Data Collection	
Microscope	Titan Krios (Thermo Fisher Scientific)
Camera	K2 Summit (Gatan)
Acceleration voltage (kV)	300
Defocus range (µm)	-0.8 to -2.5
Dose rate ($e^-/Å^2/s$)	3.33
Number of movie frames	40
Exposure time (s)	12
Total electron dose ($e^-/Å^2$)	40
Calibrated Pixel size (Å)	1.04
Reconstruction	
Box size (pixel)	200
Inter box distance (Å)	14
Number of extracted segments	103,663
Number of segments after 2D classification	103,552
Number of segments after 3D classification	70,624
Resolution, 0.143 FSC criterion (Å)	2.97
Map sharpening B-Factor ($Å^2$)	-50
Helical rise (Å)	4.825
Helical twist (°)	-1.19

Supplementary Table 3.

Structural statistics of model building and refinement.

Model parameter	***Value***
Model resolution (Å)<br><br>FSC threshold 0.143	2.97<br><br>2.97
Model resolution range (Å)	208 - 2.97
Map sharpening B factor (Å2)	0
Model composition<br><br>Non-hydrogen atoms<br><br>Protein residues<br><br>Ligands	 <br>7909<br><br>1243<br><br>-
B factors (Å2)<br><br>Protein<br><br>Ligand	 <br>89.07<br><br>-
R.m.s. deviations<br><br>Bond lengths (Å)<br><br>Bond angles (°)	 <br>0.006<br><br>1.025
Validation<br><br>MolProbity score<br><br>Clashscore<br><br>Poor rotamers (%)<br><br>CaBLAM outliers (%)	 <br>2.3<br><br>27.3<br><br>0.1<br><br>6.6
Ramachandran plot<br><br>Favored (%)<br><br>Allowed (%)<br><br>Disallowed (%)	 <br>94.5<br><br>5.5<br><br>0
